# Regulation of Unperturbed DNA Replication by Ubiquitylation

**DOI:** 10.3390/genes6030451

**Published:** 2015-06-25

**Authors:** Sara Priego Moreno, Agnieszka Gambus

**Affiliations:** School of Cancer Sciences, University of Birmingham, Vincent Drive, B15 2TT, Birmingham, UK

**Keywords:** DNA replication, ubiquitylation, proteasomal degradation, re-replication, termination, posttranslational modification, replisome

## Abstract

Posttranslational modification of proteins by means of attachment of a small globular protein ubiquitin (*i.e.*, ubiquitylation) represents one of the most abundant and versatile mechanisms of protein regulation employed by eukaryotic cells. Ubiquitylation influences almost every cellular process and its key role in coordination of the DNA damage response is well established. In this review we focus, however, on the ways ubiquitylation controls the process of unperturbed DNA replication. We summarise the accumulated knowledge showing the leading role of ubiquitin driven protein degradation in setting up conditions favourable for replication origin licensing and S-phase entry. Importantly, we also present the emerging major role of ubiquitylation in coordination of the active DNA replication process: preventing re-replication, regulating the progression of DNA replication forks, chromatin re-establishment and disassembly of the replisome at the termination of replication forks.

## 1. Introdution 

**Figure 1:**
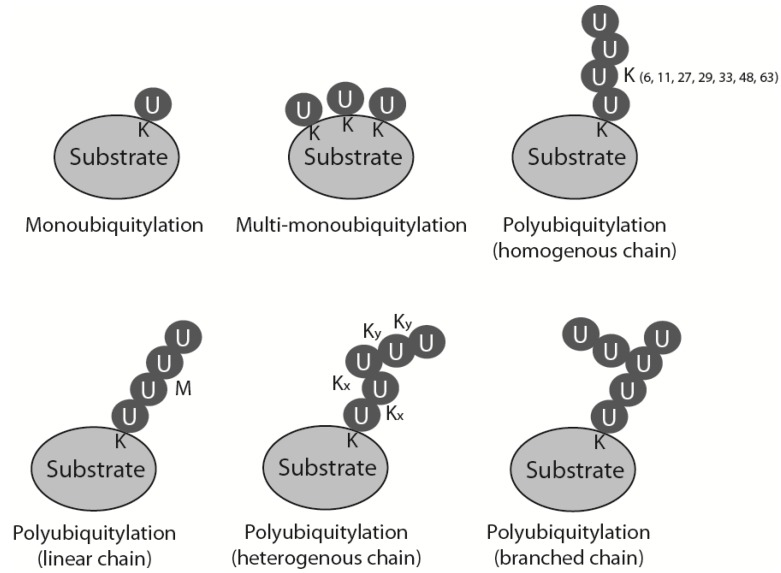
Types of substrate ubiquitylation. Mono-, multimono- and polyubiquitylation is presented. Different types of polyubiquitin chains: linked through specific but the same lysine throughout the chain (homogenous chains), linked through N-terminal methionine (linear chains), linked through alternative lysines (heterogenous chains) and chains with one of the chain’s ubiquitin modified by two further ubiquitins linked through different lysines (branched chains).

During ubiquitylation, a small (76 amino acid) polypeptide, ubiquitin, is attached covalently to the substrate protein. The ubiquitin sequence and its three-dimensional structure are highly conserved through all eukaryotes, which allows the whole system of ubiquitin attachment and signalling to be very well preserved throughout evolution. Ubiquitin contains a conserved C-terminal glycine that is attached by an isopeptide bond, usually to a lysine residue in the substrate protein. This reaction is catalysed by a cascade of three enzymes: a ubiquitin activating enzyme (E1) passes activated ubiquitin to a ubiquitin conjugating enzyme (E2), which can attach it to the substrate, usually with the help of a ubiquitin ligase (E3) [1]. Substrates can be modified with ubiquitin in many ways: they can be monoubiquitylated, multimonoubiquitylated or polyubiquitylated (Figure 1). Polyubiquitylation, *i.e.*, attachment of a ubiquitin chain to the substrate, is possible as ubiquitin contains seven lysine residues within its sequence (K6, K11, K27, K29, K33, K48 and K63) that can be used for further ubiquitin attachment and formation of ubiquitin chains. Depending on which lysine within ubiquitin is modified to form the chain, homogenous polyubiquitin chains can exhibit seven different linkages (all ubiquitins linked through the same lysine position in each ubiquitin), as well as: linear chains linked through its N-terminal methionine, mixed heterogenous linkage chains and even branched structures (Figure 1) [1]. Attachment of ubiquitin or a ubiquitin chain to the substrate changes the overall three-dimensional structure of the substrate and affects the substrate’s activity, localization and fate. Each type of chain has its own unique three-dimensional structure and thus constitutes a different signal and a different outcome for the substrate. The best-studied forms of ubiquitylation are observed when a protein is modified with K48 and K11 linked chains that targets it for proteasomal degradation, and modification with K63 linked chains which plays a crucial role in DNA damage response (DDR) signalling [2]. Ubiquitin can also be removed from the substrate or edited through action of the de-ubiquitylating enzymes (DUBs). Ubiquitylation as a posttranslational modification is, therefore, very flexible and versatile. It is also one of the most abundant types of protein modification in the cell [1].

The essential role for ubiquitylation in the regulation of the DDR and replicative stress processes has been studied for years [2], but its importance in regulation of unperturbed DNA replication has been established more recently. In this review, we will focus on describing the role of ubiquitylation specifically during unperturbed DNA replication. We will summarise the role for ubiquitin-driven degradation in origin licensing and entry into S-phase, discuss its importance in blocking re-replication during S-phase and chromatin re-establishment and finally focus on recent findings in the regulation of replisome factors during replication elongation and termination.

## 2. Setting the Scene for Origin Licensing

**Figure 2:**
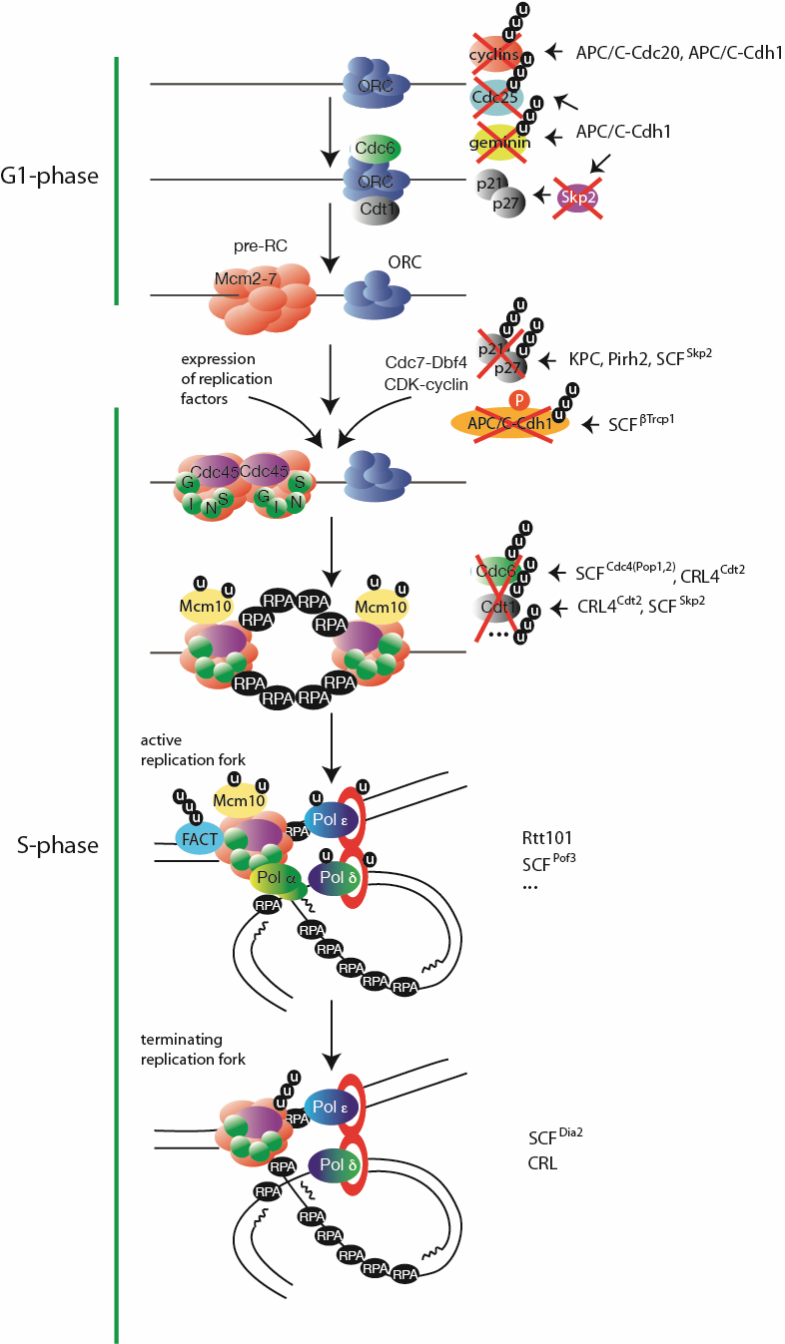
Ubiquitylation regulates every stage of eukaryotic DNA replication. A simplified view of steps in DNA replication with indicated major substrates of ubiquitylation regulation. On the right hand side the respective ubiquitin ligases are listed, if known. Further explanations can be found in the text.

The licensing of replication origins occurs before the onset of S-phase: at the end of mitosis and through the G1-stage of the cell cycle. During licensing Origin Recognition Complex (ORC), Cdt1 and Cdc6 load multiple Mcm2-7 complexes onto origins forming pre-replicative complexes (pre-RCs) (reviewed in [3]). This loading reaction requires a low activity of Cyclin Dependent Kinases (CDK). Therefore, to allow licensing, the high CDK activity of mitotic CDKs needs to be abolished. The low CDK activity in G1-phase of cell cycle is achieved through (i) degradation of mitotic cyclins, (ii) degradation of CDK activator: tyrosine phosphatase Cdc25 and (iii) accumulation of CDK inhibitors (CKIs). Both mitotic cyclins and Cdc25 are degraded in a proteasome dependent manner upon their ubiquitylation by a master cell cycle regulator: the Anaphase Promoting Complex or Cyclosome (APC/C) [4]. APC/C is a multisubunit ubiquitin ligase that polyubiquitylates proteins with K48 and K11 linked ubiquitin chains, targeting them for proteasomal degradation [5]. APC/C utilises two substrate recognising adaptor proteins, Cdc20 and Cdh1 [6], which target proteins containing specific recognition motifs: D-boxes and KEN-boxes [7]. Cdc20 binds APC/C during mitosis in a CDK phosphorylation dependant manner [8] and is responsible for the initial degradation of mitotic cyclins (and other mitotic proteins) [9]. As the level of cyclins drops, the mitotic phosphatase Cdc14 has a chance to dephosphorylate and activate Cdh1, which can then compete with Cdc20 for binding to APC/C [10]. APC/C-Cdh1 has a vast repertoire of substrates, and importantly leads to ubiquitylation and degradation of Cdc20, further decline of mitotic cyclins, degradation of the Cdc25 phosphatase (CDK activator) and progression to G1-phase [11,12]. APC/C-Cdh1 also allows accumulation of CDK inhibitors: CKIs and the INKa family of proteins (p15, p16, p17 and p19) [13,14]. CKIs accumulate as APC/C promotes degradation of Skp2—a substrate receptor of ubiquitin ligase ubiquitylating CKIs for degradation [13,15] (Figure 2).

Apart from ensuring that the CDK activity is abolished to allow licensing of origins, APC/C-Cdh1 also triggers the accumulation and activity of licensing factors in G1-phase. In metazoans, APC/C-Cdh1 regulates the stability of the large subunit of the origin recognition complex, Orc1 [16], Cdc6 [17] and targets Cdt1 inhibitor geminin for degradation [18]. Altogether, APC/C activity creates favourable conditions for origin licensing by eliminating cyclins and, in metazoans, geminin (Figure 2).

## 3. Initiation of DNA Replication

**Figure 3:**
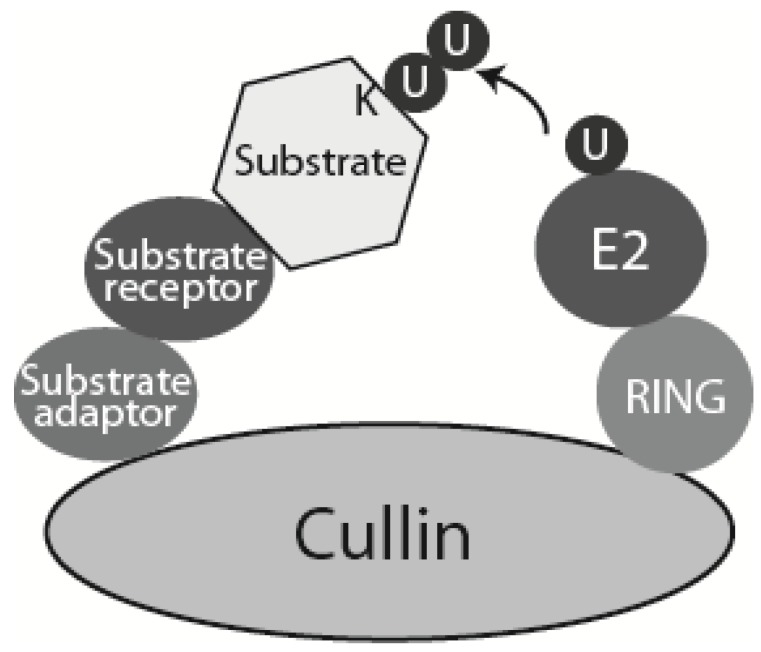
Schematic model of CRLs. Cullin-RING ligases (CRLs) are multisubunit ubiquitin ligases built around a scaffold cullin subunit, which interacts at its C-terminus with RING domain ubiquitin ligase subunit Rbx1 or Rbx2, and at its N-terminus with substrate adaptor and substrate receptor. Higher eukaryotes express seven different cullins (Cul1, 2, 3, 4A, 4B, 5 and 7). Each of them interacts with a specific set of substrate adaptor and receptor proteins. In case of SCFs (Cullin 1 based CRL: Skp1-Cullin 1-F box) the substrate adaptor is Skp1 and substrate receptor is one of many F-box containing proteins [22]. CRL4 contains Cullin 4, Ddb1 as substrate adaptor and DCAF domain protein as substrate receptor.

Initiation of DNA replication requires increased activity of the S-phase kinases (CDK and Cdc7) and expression of cell-cycle regulated initiation factors. To allow the rise in CDK level, CDK inhibitors have to be degraded and APC/C-Cdh1 activity diminished. Both are mainly driven through ubiquitylation directed proteasomal degradation and require activation of G1-CDKs at the mammalian G1 “restriction point” (“START point” in yeast), which is possible as G1 cyclins are not generally APC/C substrates. In mammalian cells, Cdk4 and Cdk6 are involved in early G1, while Cdk2 is believed to complete G1 and initiate S-phase. Positive growth stimuli in G1 induce expression of Cyclin D, which, together with Cdk4 and Cdk6, activates a programme of gene expression of S-phase factors. Key regulators of this programme are: the Retinoblastoma protein family (Rb), transcription factors E2F1-3 and Myc (reviewed in [19]). This transcriptional programme induces expression of Cyclin E-Cdk2, which is inactive through most of G1 due to high levels of CKIs (p27 and p21) [20]. In mammalian cells, p27 is ubiquitylated and degraded by three ubiquitin ligases: (i) KPC ligase targets p27 exported from the nucleus to the cytoplasm in G0 and G1-phases; (ii) Pirh2, whose expression increases from G1 to S-phase; and (iii) SCF^Skp2^, which from early S-phase, targets nuclear p27 (reviewed in [21]). SCF (Skp1-Cullin1-Fbox) belongs to the cullin-RING ligase (CRL) family of multisubunit ubiquitin ligases (Figure 3) [22]. The F-box substrate receptor Skp2 of the SCF^Skp2^ ubiquitin ligase specifically recognises Thr187-phosphorylated p27, which is modified by any Cyclin E-Cdk2 that escaped CKI inhibition [23]. SCF^Skp2^ also targets another CKI, p21, for proteasomal degradation (reviewed in [21]) (Figure 2).

Apart from degrading CKIs, APC/C-Cdh1 activity has to be blocked to allow S-phase entry. This happens in a number of ways: (i) Cdh1 is phosphorylated by Cdk2-Cyclin E, which blocks Cdh1 activity [24]; (ii) Cdh1 is phosphorylated by Polo-like kinase Plk1 and Cdk2-Cyclin A, which directs it for polyubiquitylation by another ubiquitin ligase, SCF^βTrcp1^ [25]; (iii) accumulation of the APC/C-Cdh1 inhibitor Emi1 [26]; and (iv) finally APC/C-Cdh1 can autoubiquitylate itself using the E2, UbcH10 [27]. As a result, the transition from G1 to S-phase brings the end of the reign of APC/C ubiquitin ligase and the beginning of rule by CRL ubiquitin ligases (Figure 2).

Once cells enter S-phase they use a wide number of SCF ligases (CRL1) to ubiquitylate and degrade factors essential for G1 to S-phase transition but are now dispensable or detrimental. SCF^FBX4^ promotes the degradation of Cyclin D [28], SCF^Fbw7^ ubiquitylates Cyclin E [29], SCF^Skp2^ promotes the degradation of E2F [30], while c-Myc abundance is controlled by two SCF-type E3 ligases—SCF^Skp2^ and SCF^Fbw7^ [21].

Another CRL ubiquitin ligase that plays a key role during S-phase is a Cullin 4 based ligase CRL4 (Figure 3) (reviewed in [31,32]). CRL4 acts in a PCNA-dependent ubiquitylation pathway. PCNA is a clamp loaded onto DNA to increase processivity of DNA polymerases at the replication forks. In addition to this role, PCNA creates an organising centre and a platform for multiple factors interacting with replication forks. Some of the PCNA interacting proteins (PIPs) are substrates of the CRL4^Cdt2^ ubiquitin ligase. Proteins interacting with PCNA most often contain a short PCNA binding motif (PIP box) [33], while CRL4^Cdt2^ substrates contain not only a PIP box but also four amino acids downstream of the PIP box, a “B + 4” basic residue (PIP + TD motif, or PIP-degron), essential for recruitment of CRL4^Cdt2^ to PCNA bound substrate [34]. PCNA itself also plays an active role in recruitment of CRL4^Cdt2^ to chromatin [35].

The first *bona fide* substrate of CRL4^Cdt2^ identified was Spd1—the fission yeast inhibitor of ribonucleotide reductase (RNR) enzyme that catalyzes the synthesis of dNTPs [36]. Degradation of Spd1 in S-phase is the key function of CRL4^Cdt2^ in *S.pombe* and a lack of this degradation activity leads to DNA mutagenesis, slow growth and inhibition of double strand break repair [37].

In *Drospohila*, the transcription factor E2f1 promotes transcription of genes in G1 whose expression is essential to enter S-phase. Once cells enter S-phase, E2f1 is degraded by CRL4^Cdt2^ [38]. *Drosophila* E2f1 is unique in the fact that it contains a PIP + TD motif, and thus alternative destruction pathways are utilised in other organisms [39].

The most recently discovered substrate of CRL4^Cdt2^ is a uracil DNA glycosylase (TDG) involved in the base excision repair pathway [40]. In human cells, this enzyme accumulates during G2-M and G1 phases, while its levels are compromised during S-phase in a proteasomal degradation dependent manner [41]. TDG has a conserved PIP + TD degron motif and inhibition of TDG degradation slows down S-phase progression and leads to an increase in γH2AX foci and DNA breaks [42]. Consistent with this, it has been also shown that TDG is destroyed during S-phase in early *Xenopus* development [43].

The most striking role of CRL4^Cdt2^ is, however, the degradation of origin licensing factors to prevent re-replication during S-phase, as described below [31,32].

## 4. Blocking Re-Replication

Upon initiation of DNA replication it becomes essential that no more origins of replication can be licensed to avoid re-licensing already replicated DNA and re-replication. Replication of the same DNA twice is likely to lead to chromosomal instability and cell death [44]. To avoid re-replication, cells degrade or inhibit the activity of origin licensing factors upon entering S-phase. In general, all organisms use multiple overlapping mechanisms to prevent DNA re-replication, but the choice of pathways differs significantly between species. Here we will focus on the ones dependent on ubiquitin-mediated proteolysis.

*S. pombe* and vertebrates degrade Cdt1 in a PCNA-dependent manner through ubiquitylation by CRL4^Cdt2^ [45]. Mammalian cells degrade Cdt1 also through an additional mechanism—utilising SCF^Skp2^ [46,47]. The interaction of Cdt1 and Skp2 requires phosphorylation of Cdt1 by S-phase CDKs, providing S-phase specificity of this degradation [47]. Both of the above pathways overlap in mammalian cells and thus mutation of one pathway motifs is not sufficient to inhibit Cdt1 degradation, but deletion of the N-terminal part of Cdt1 (which contains all regulatory elements for both pathways) results in stable Cdt1 [48,49,50]. Finally, any Cdt1 remaining through S-phase until G2 can be ubiquitylated by SCF^Fbx°31^ and degraded [51] (Figure 2).

Budding yeast Cdc6 and its fission yeast homolog Cdc18 are degraded in S-phase, upon their phosphorylation by CDK, through ubiquitylation by SCF^Cdc4^ and SCF^P°p1,2^, respectively [52,53,54]. In human cells, Cdc6 activity is inhibited mainly through CDK stimulated nuclear export in S-phase [55,56], but a recent report suggests a degradation of Cdc6 through CRL4^Cdt2^ pathway due to a PIP box within Cdc6 [57]. Finally, ubiquitylation and degradation of the CDK inhibitor p21, by CRL4^Cdt2^ and SCF^Skp2^, blocks re-replication as it allows full activity of S-phase CDKs, leading to CDK driven phosphorylation of Cdc6 and its nuclear export [56,58].

The Orc1-6 complex is regulated in different ways throughout evolution: mostly the levels of Orc1-6 remain stable throughout the cell cycle. It has been reported, however, that in transformed human cells the majority of Orc1 can be selectively degraded during S-phase in a proteasomal dependent manner, most likely through SCF^Skp2^ ubiquitin ligase activity [59,60]. The Orc1-6 interacting partner ORCA, which plays a role in origin licensing in human cells, has also been shown to be ubiquitylated by CRL4A(Ddb1) at the onset of S-phase, but the role of this ubiquitylation is not yet clear [61]. Finally, interaction of the Orc1-6 complex with chromatin and licensing ability of the cell depends on chromatin structure and epigenetic marks, *i.e.*, Orc1 binds to methylated histones H3 and H4 [62]. The degradation of the enzyme responsible for these modifications: histone methyl-transferase Set8, in a PCNA- and CRL4^Cdt2^-dependent manner, also contributes to blocking re-replication, while non-degradable Set8 mutant causes uncontrolled re-replication [63,64].

Taking into account the key and widespread role of CRL4^Cdt2^ in blocking origin licensing, it is not surprising that its activity is highly controlled in order to restrict its activity to S-phase. This control is executed not only through the fact that PCNA needs to be chromatin bound to stimulate substrate recognition, but also through the modulation of Cdt2 turnover. In human cells, Cdt2 is ubiquitylated and degraded by two mechanisms: by SCF^Fbx°11^ and by PCNA-independent autoubiquitylation by CRL4A [65]. To prevent Cdt2 from being ubiquitylated in S-phase, it is phosphorylated by Cyclin B-Cdk1, which inhibits its interaction with SCF^Fbx°11^ [65,66,67].

## 5. Ubiquitylation of Replisome Components during the Elongation Stage of DNA Replication

The progression of replication forks can be affected by a number of different post-translational modifications to the replisome factors. Moreover, a whole raft of modifications is applied when forks encounter DNA damage (reviewed in [68]). We will focus here on the ubiquitylation events regulating replisome components during unperturbed DNA replication (Figure 2).

In all eukaryotic systems examined to date, PCNA undergoes monoubiquitylation of a highly conserved Lys164 (K164) by the RAD18-RAD6 ubiquitin ligase in response to replication block (reviewed in [69]). Monoubiquitylation of K164 on PCNA plays a key role in promoting the interaction of PCNA with Y-family translesion DNA synthesis (TLS) of DNA polymerases and lesion bypass. Sometimes, this monoubiquitylation can be extended to create a K63 linked ubiquitin chain. Such polyubiquitylation of PCNA promotes error-free repair of the lesion involving template switch DNA synthesis. Other lysines within PCNA can also be ubiquitylated upon DNA damage, but the role of such ubiquitylation is less well-defined [69]. However, in *Xenopus laevis* egg extract a proportion of PCNA can be ubiquitylated constitutively on K164 during unperturbed DNA replication. This ubiquitylation is important for efficient DNA replication and its inhibition leads to reduction of the level of lagging strand polymerase, Pol∂, associated with chromatin [70].

Another replisome factor ubiquitylated during normal S-phase is Mcm10. Mcm10 has been shown to play important roles during both the initiation and the elongation stages of DNA replication. Although there is some controversy in the field, the current consensus is that Mcm10 is loaded onto chromatin after origin licensing and is needed for the activation of the replicative helicase [71]. Mcm10 has been also found to interact with and maintain the stability of DNA polymerase α (priming polymerase) as well as its association with chromatin [72]. Sapna Das-Bradoo *et al* showed that Mcm10 is monoubiquitylated at two distinct lysine residues during G1-S phase, and this modification allowed the interaction of ubiquitylated Mcm10 with PCNA. This interaction event has been considered of essential importance during DNA replication since its impairment compromised cell growth [73]. The fact that ubiquitylated Mcm10 is unable to interact with Polα raises the possibility that this modification of Mcm10 could promote the release of Polα after the completion of the RNA-DNA primer synthesis and the recruitment of PCNA [71,73].

Another polymerase affected by ubiquitylation is the lagging strand polymerase, Pol∂. Pol∂ is formed of 4 subunits: the catalytic p125 and regulatory p50, p66 and p12. Both p12 and p66 have been shown to be ubiquitylated (predominantly monoubiquitylated) in human U2OS osteosarcoma cells in unperturbed S-phase [74]. Neither of these ubiquitylations leads to proteasomal degradation of the subunits - it is more likely that they can instead regulate protein-protein interactions [74].

A study performed in *S. pombe* showed also that Pol2 (a catalytic subunit of the leading strand polymerase, Polε) is continuously degraded by the proteasome during unperturbed S-phase in a manner dependent on the ubiquitin ligase SCFPof3 - an effect clearly visible especially in cells harbouring Swi1 deletion and thus experiencing replisome stability problems. However, the level of Pol2 in the cells is constant due to continuous Pol2 translation. The authors suggested that this degradation mechanism could serve to “refresh” Pol2 enzymes during S-phase: provide newly synthesised Pol2 enzyme to be reloaded onto the leading strand [75,76].

It has also been reported that *S.cerevisiae* Spt16, a subunit of the FACT complex (FAcilitates Chromatin Transcription), is a substrate of the E3 ubiquitin ligase complex containing Rtt101 [77]. FACT, a heterodimeric complex of Spt16 and SSRP1, has nucleosome remodeling activity, which makes it an important player during transcriptional regulation [78]. Apart from this, FACT has been found to be an integral component of the Replisome Progression Complex (RPC) in budding yeast [79] and an interaction partner of the MCM complex [80]. Ubiquitylation of Spt16 does not target it for proteasomal degradation but it has been proposed to play a role in the association of FACT with replication origins and MCM complexes. Moreover, it was suggested that it promotes the recruitment of MCM complexes to replication origins during G1 and early S-phase. Interestingly, the impairment of Spt16 ubiquitylation does not affect the role of FACT in transcription, which suggests that this mechanism specifically regulates FACT function during DNA replication [77].

## 6. Chromatin Re-Establishment during DNA Replication

While DNA is replicated, the chromatin structure becomes locally disassembled ahead of the replication forks and re-assembled behind the forks. This process involves eviction of nucleosomes in front of the fork by fork-associated histone chaperones: FACT and Asf1, followed by their re-assembly on newly formed sister chromatids. The parental histones are then joined by newly synthesised ones to provide enough nucleosomes to organise the doubled quantity of DNA [81]. As the new nucleosomes do not contain the histone modifications present on the parental nucleosomes these modifications have to be reconstituted behind the fork to provide the daughter cells with the same epigenome profile. The present model implies that histone-modifying enzymes can recognise the modifications on parental nucleosomes and replicate them on the neighboring, new nucleosomes. DNA replication also produces hemimethylated DNA, as the newly synthesised DNA strand contains unmodified cytosines. In this case, DNA methyltransferase 1 (Dnmt1) methylates the hemimethylated fragments of DNA behind the fork to produce a DNA strand identical to the parental one [82]. Importantly to this review, ubiquitylation plays a key role in many aspects of this chromatin reconstitution process.

Firstly, it is important that the expression and translation of histones is tightly regulated and coupled to the DNA replication process. It has been shown that the lack of proper regulation of histones levels during the cell cycle leads to defects in chromosome transmission fidelity and genomic instability [83,84]. The synthesis of histones is regulated by ubiquitin driven degradation of their transcription factors. In fission yeast, Ams2—a transcription factor responsible for core histone gene expression, is degraded in G1 by APC/C-Cdh1 [85] and after S-phase by SCF^P°f3^ [84]. This results in the synthesis of histones only in S-phase.

It was shown also that levels of “free” (non-chromatin bound) histones are regulated directly by ubiquitin driven proteasomal degradation. In budding yeast, non-chromatin bound histones were found to be degraded with a half-life of 30–40 min [86] in a manner dependent on the activity of the checkpoint kinase Rad53. The mechanism employs Ubc4/5 E2 enzymes and Tom1, Pep5, Snt2, Hel1 and Hel2 ubiquitin ligases [87,88]. Interestingly, the human homologue of Tom1: HUWE1, has also been reported to be able to ubiquitylate all four core histones *in vitro* [89].

The assembly of histones into nucleosomes on DNA is also regulated by ubiquitylation; specifically the deposition of newly synthesized histone H3 onto replicated DNA. Budding yeast Rtt101^Mms1^ E3 ubiquitin ligase preferentially ubiquitylates new histone H3 acetylated at lysine 56 (H3K56Ac-H4) *in vitro* and *in vivo*. This ubiquitylation of histone H3 at K121, 122 and 125 leads to release of H3-H4 from Asf1 to other histone chaperones followed by their incorporation into chromatin [90]. The human orthologue of Rtt101^Mms1^, Cul4A^DDB1^ appears to have an analogous role, since human cells depleted of Cul4A^DDB1^ activity show a similar defect in H3 deposition [90].

Another histone ubiquitylation event that regulates unperturbed DNA replication is the monoubiquitylation of histone H2B (H2Bub1) driven by the E3 ligase Bre1. A key role for H2Bub1 is to control the transcription of genes through regulation of Pol II elongation [91]. Transcription-independent functions of H2Bub1 include roles in meiosis, the UV-induced checkpoint, DNA double-strand breaks repair, apoptosis and Set1-directed methylation of the kinetochore associated protein Dam1 [92,93,94,95]. Moreover, H2Bub1 also coordinates activation of the intra-S checkpoint and chromatin assembly during replication under stress [96]. In budding yeast, H2Bub1 (on lysine 123) has been shown to occur on chromatin adjacent to replication origins localized at non-transcribed regions. This ubiquitylation event was found to be required for appropriate nucleosome assembly or nucleosome stability during DNA replication in times of replication stress (in the presence of hydroxyurea, HU). Under these conditions, impairment of H2B ubiquitylation also led to a defect in replication fork progression and replisome stability. Importantly however, during unperturbed DNA replication, *htb-K123R* cells displayed a delay in the completion of DNA replication implicating the important role of the H2Bub1 in normal replication processes [96].

Finally, a recent study suggested that Uhrf1 dependent polyubiquitylation of histone H3 on lysine 23 (H3K23nUb) serves as a platform for Dnmt1 to bind the hemimethylated DNA behind the replication fork in order to fully methylate it [97]. Uhrf1 is an ubiquitin ligase which can bind hemimethylated DNA through its SRA domain and has also been shown to interact with PCNA [98,99]. These two interactions bring Uhrf1 to the replicating fork where it ubiquitylates H3, which in turn is preferentially bound by Dnmt1. Interestingly, due to a negative feedback loop (once DNA is methylated, H3K23nUb is deubiquitylated), the polyubiquitylated form of H3 could be detected only upon inhibition of Dnmt1 activity [97].

## 7. Termination of Replication Forks

Recent studies in budding yeast and *Xenopus laevis* egg extract have shown that polyubiquitylation plays a key role in disassembly of the replisome machinery at the termination of DNA replication forks [100,101]. In both model systems, only one subunit of the replicative helicase—Mcm7—has been found polyubiquitylated with K48 linked chains upon replication fork termination (Figure 2). This ubiquitylation was then followed by dissolution of the replicative helicase (CMG complex), which is dependent on the activity of the protein segregase, Cdc48/p97/VCP. The segregase is a homohexameric ring-shaped ATPase, which can recognise proteins modified with K48 linked ubiquitin chains and remodel them in an ATP-dependent manner [102,103]. Such remodelling can lead to removal of the ubiquitylated protein from the endoplasmic reticulum membrane (ERAD—endoplasmic-reticulum-associated protein degradation) or chromatin [104,105]. The ubiquitylated protein can then be directed for proteasomal degradation or deubiquitylated by one of the DUBs interacting with the segregase [106]. The fate of ubiquitylated Mcm7 upon its recognition and remodelling by the segregase has not yet been established.

In budding yeast, Mcm7 is ubiquitylated by SCF^Dia2^ ubiquitin ligase [100], while the ligase in higher eukaryotes is not yet known, although the ubiquitylation process in *Xenopus* egg extract is dependent on the CRLs [101]. SCF^Dia2^ has been previously shown to interact with the replisome and travel with the replication fork with the RPCs [79,107]. Nevertheless, SCF^Dia2^ recognises only the terminated helicase for ubiquitylation, and not the active form. In cells lacking Dia2 the replicative helicase complexes persist on chromatin until the subsequent cell cycle [100] and at the same time, Dia2Δ cells exhibit a high level of genomic instability and have problems with replicating through difficult-to-replicate DNA regions [108,109]. Dia2 was also shown to accumulate when the intra-S-phase checkpoint was activated, and to promote ubiquitylation of Mrc1 leading to its degradation [109,110]. Clearly, SCF^Dia2^ is likely to have many functions at the replication forks.

## 8. Concluding Remarks

Recent years have brought a small explosion of examples of substrates and mechanisms through which ubiquitylation regulates the process of unperturbed DNA replication. With this knowledge a multitude of questions arises about the regulation, fine-tuning and coordination of all of these ubiquitin dependent mechanisms. Many of the enzymes involved also have additional functions upon replication stress, bringing another level of complexity to the emerging ubiquitin regulation network.

It is tempting to speculate about whether the emerging network of ubiquitylation enzymes, essential for faultless execution of DNA replication, could lead to the next generation of cancer therapy targets [111]. Many of the traditional therapies target DNA replication and DNA repair processes but they tend not to be cancer specific and thus exhibit a restricted therapeutic window due to side effects. Many of the enzymes involved in the ubiquitin regulatory network are deregulated in cancer, providing the opportunity to design and use drugs targeted against them [112]. One example of such a potential drug is MLN4924—a small molecule inhibitor of the Nedd8 activating enzyme, which blocks activity of the cullin-RING ubiquitin ligases CRLs. Treatment of variety of human tumour cell lines with MLN4924, results in uncontrolled DNA synthesis through re-replication, leading to DNA damage and induction of apoptosis [113,114]. Importantly, the potential of antitumor activity was shown in human colon and lung xenograft models at doses and schedules that were well tolerated and which led to multiple phase I trials of MLN4924 [113,115]. Future research will establish a better understanding of the ubiquitin regulation of DNA replication and the opportunity to use it to treat cancer.
